# 

**Published:** 1853-09

**Authors:** 


					﻿RECEIPTS
FOR TllE JOURNAL DURING THE MONTH OF AUGUST.
Illinois.
D',	E. T. Cbapaian, -	•	$4	00
”	E. Bennett, -	-	-	-	2	00
”	W. I’. H.'ilvii,	6	00
”	H K. Lithv, -	-	-	-	2	0)
”	W W. Welch,	2	"0
” D ick..............................0)
”	.1. W M'Kenney,	-	•	4	OJ
”	E.	E. Webster,	-	-	-	2	00
”	Jeremiah H. Lyfford,	•	-	4	oO
”	A.	W. Bentnn.	-	-	-	2	0)
”	l.a'ti Wallace. -	•	-	2	CO
”	(J.	W. Southwick,	•	-	-	2	00
”	S A Pi I lock, ...	5 00
”	.1. V. (ioltra, ....	200
”	A. Malian, -	-	-	-	2 uO
O ii io.
Dr A. W. D wey, -	-	-	4 0J
W ISCONSIN.
Dr. E. G Dyer, ...	2 Of)
”	A. s Castleman,	.	-	-	2 00
” James Prentice, - •	2 00
”	S. U, Johnson.	-	-	•	2 00
” J.1I.& A. B. Wright, -	3 00
Iowa.
Dr. w. D. Nelson,	-	-	-	2 03
” E. M Downs, ...	2 00
9	Indiana.
Dr, Sheldon. ....	4	00
” .TH TFilev. -	-	-	-	2	00
”	A. B. Buch unn.	-	-	-	2	0J
”	shirlev A Marshall, -	-	4	00
”	S. B. Kiler.	...	4	00
”	J. E. Hendrick, -	-	-	2	00
■’	O II M etin,	...	2	00
” John i.e'v s, ...	-2	0J
” Jas. A. W >od, ---	2 00
” W. II. Giff' d. -	-	-	2 00
”	Tims. W, M ille.L -	-	2	0j
” C. II- P. 1,. Evans, - -	4 00
”	R. I). M i izy. ...	2	00
” Sami! Kelly, - - - - 2 Oj
”	R. A. Perkins, -	-	-	4	Oj
Missouri.
Dr. C. M. Smith, -	-	-	4 00
New York.
Dr. T. R. Beck, -	-	-	4 03
” D.E. Ellis, -	-	-	-	2 00
Michigan.
Dr. Lovell,..........................2	oo
” Z. Theirs Slater, -	-	2 Oo
”	.1 S kinner,	...	2 00
” M. Adams, ...	2 00
				

